# Quantitative Susceptibility Mapping in Parkinson's Disease

**DOI:** 10.1371/journal.pone.0162460

**Published:** 2016-09-06

**Authors:** Christian Langkammer, Lukas Pirpamer, Stephan Seiler, Andreas Deistung, Ferdinand Schweser, Sebastian Franthal, Nina Homayoon, Petra Katschnig-Winter, Mariella Koegl-Wallner, Tamara Pendl, Eva Maria Stoegerer, Karoline Wenzel, Franz Fazekas, Stefan Ropele, Jürgen Rainer Reichenbach, Reinhold Schmidt, Petra Schwingenschuh

**Affiliations:** 1 Department of Neurology, Medical University of Graz, Graz, Austria; 2 Medical Physics Group, University Hospital-Friedrich Schiller University Jena, Jena, Germany; 3 Buffalo Neuroimaging Analysis Center, Department of Neurology, University at Buffalo, The State University of New York, Buffalo, NY, United States of America; 4 MRI Molecular and Translational Imaging Center, Clinical and Translational Research Center, University at Buffalo, The State University of New York, Buffalo, NY, United States of America; 5 Michael Stifel Center for Data-driven and Simulation Science Jena, Friedrich Schiller University Jena, Jena, Germany; 6 Division of Neuroradiology, Department of Radiology, Medical University of Graz, Graz, Austria; Universitat Ulm, GERMANY

## Abstract

**Background:**

Quantitative susceptibility mapping (QSM) and R2* relaxation rate mapping have demonstrated increased iron deposition in the substantia nigra of patients with idiopathic Parkinson’s disease (PD). However, the findings in other subcortical deep gray matter nuclei are converse and the sensitivity of QSM and R2* for morphological changes and their relation to clinical measures of disease severity has so far been investigated only sparsely.

**Methods:**

The local ethics committee approved this study and all subjects gave written informed consent. 66 patients with idiopathic Parkinson’s disease and 58 control subjects underwent quantitative MRI at 3T. Susceptibility and R2* maps were reconstructed from a spoiled multi-echo 3D gradient echo sequence. Mean susceptibilities and R2* rates were measured in subcortical deep gray matter nuclei and compared between patients with PD and controls as well as related to clinical variables.

**Results:**

Compared to control subjects, patients with PD had increased R2* values in the substantia nigra. QSM also showed higher susceptibilities in patients with PD in substantia nigra, in the nucleus ruber, thalamus, and globus pallidus. Magnetic susceptibility of several of these structures was correlated with the levodopa-equivalent daily dose (LEDD) and clinical markers of motor and non-motor disease severity (total MDS-UPDRS, MDS-UPDRS-I and II). Disease severity as assessed by the Hoehn & Yahr scale was correlated with magnetic susceptibility in the substantia nigra.

**Conclusion:**

The established finding of higher R2* rates in the substantia nigra was extended by QSM showing superior sensitivity for PD-related tissue changes in nigrostriatal dopaminergic pathways. QSM additionally reflected the levodopa-dosage and disease severity. These results suggest a more widespread pathologic involvement and QSM as a novel means for its investigation, more sensitive than current MRI techniques.

## Introduction

The loss of dopaminergic neurons in the pars compacta of the substantia nigra is a patho-histological hallmark of Parkinson’s disease (PD), which is paralleled by increased iron deposition [1,2]. Iron in the human brain is essential for numerous biological processes such as myelination, DNA synthesis, and mitochondrial respiration [3]. However, iron dysregulation can exacerbate deleterious excess formation of radical species and can induce oxidative stress by the Fenton reaction [4]. Although abnormally high iron deposition in deep gray matter is a frequent but rather unspecific finding observed in neurodegenerative diseases, there is an increasing interest in iron chelation therapy and therefore its reliable and accurate measurement in vivo [5].

While early investigations about iron deposition were limited to histology, the advent of MR imaging into clinical research allows to assess iron concentration also in longitudinal study designs [6]. While with clinical MR scans elevated iron levels can be deduced from signal loss on T2-weighted and especially T2*-weighted images, newer R2 and R2* relaxation rate mapping have allowed to measure iron in a more refined and quantitative manner [7,8]. However, iron is paramagnetic and consequently it is not only increasing the relaxivity as reflected in R2 and R2*, but also shifting the magnetic susceptibility of brain tissue toward more positive values [9]. This has led to the development of a novel MR technique for the assessment of brain iron in vivo which is quantitative susceptibility mapping (QSM) [10]. QSM based iron mapping is technically more demanding than relaxation rate mapping or the rating of T2 or T2* hypointensities but comes with the benefit of higher sensitivity regarding the quantitative assessment of pathologic tissue changes [11]. Furthermore, QSM has been validated in recent postmortem studies demonstrating that magnetic susceptibility in deep gray matter is highly correlated (r = 0.84) with the iron concentration as determined by inductively-coupled plasma mass spectroscopy [12] and Perls’ iron staining [13].

Thus far, QSM has only rarely been used for the investigation of patients with PD. Very recent work found higher susceptibility in the substantia nigra [14–16], and additionally in the nucleus ruber of patients with PD [17]. However, as the aforementioned reports on an involvement of the basal ganglia are controversial [18], we here investigated this open question in a relatively large cohort of patients with PD, also including patients with a longer disease duration. The aim of this explorative study was to apply quantitative susceptibility mapping (QSM) and R2* mapping in gray matter nuclei of patients with PD, to compare the results to age-matched controls and to assess the relationship between QSM and R2* measures with clinical and morphological measures. Additionally, we investigated whether magnetic susceptibility is correlated differently with clinical parameters in patients with shorter versus those with a longer disease duration.

## Methods

### Subjects

The ethics committee of the Medical University of Graz approved this study and all subjects gave written informed consent. The study sample consisted of 66 PD patients (44 males, 22 females; mean age = 64.7 years) and 58 age-matched healthy controls (34 males, 24 females; mean age = 65.0 years). All patients were participants in the single-center cohort study “Prospective Movement Disorders Registry Graz” (PROMOVE) enrolled from our outpatient clinic between 08/2010 and 05/2013. The patients had to fulfill he following inclusion criteria: diagnosis of PD according to the UK Parkinson's Disease Society Brain Bank criteria [19], age above 45 years, a mini-mental state examination (MMSE) score ≥ 24 [20], availability of a detailed neurological as well as comprehensive MRI examination including gradient echo phase images. Healthy controls (CON) without known neuropsychiatric disorder were recruited from an ongoing community-dwelling aging cohort [21].

Characteristics of study participants are summarized in Table 1. From the total of 66 patients with Parkinson’s disease, 54 were tremor dominant (TD), 11 had postural instability/gait difficulty (PIGD), and one equivalent [22]. Patients were classified into 32 right dominant PD, 28 left dominant PD and 6 were equally affected [23].

**Table 1:** Clinical and demographic data of the study cohort.

	Controls	Parkinson’s disease	p-value
N	58	66	NA
N female (%)	23 (39%)	24 (36%)	n.s. ^a^
Age (years) †	65.0 (9.3)	64.7 (8.8)	n.s. ^a^
Disease duration (years) ††	NA	3.4 (0.25–24.9)	NA
MDS-UPDRS 1 †	NA	7.2 (4.6)	NA
MDS-UPDRS 2 †	NA	11.4 (6.1)	NA
MDS-UPDRS 3 †	NA	31.3 (14.6)	NA
MDS-UPDRS 4 †	NA	0.4 (1.8)	NA
MDS-UPDRS total †	NA	50.2 (21.5)	NA
H&Y †	NA	2.0 (0.5)	NA
LEDD †	NA	182.5 (436.9)	NA

N = number of patients/controls; MDS-UPDRS = Movement Disorder Society sponsored revision of the Unified Parkinson's Disease Rating Scale consisting of four parts; H&Y = Hoehn & Yahr scale; LEDD = levodopa equivalent daily dose; n.s. = not significant (p>0.05); NA = not applicable; values are given as frequency (percent);

^†^ mean (standard deviation) or as

^††^ = median (range);

^a^ t-test.

### Clinical Assessment of Disability

Disease duration was determined from the date of the physician-confirmed diagnosis of PD to the date of the study visit as the former date can be more precisely identified than the date of the first, PD related symptoms. Fourteen patients were drug-naïve and 52 patients were on optimized antiparkinsonian medication. The levodopa-equivalent daily dose (LEDD) was calculated for each patient [24]. Clinical examinations were performed in a practical OFF state (overnight withdrawal, i.e. off prescribed antiparkinsonian medication for at least 12 hours). Disease severity was assessed using the Hoehn & Yahr (H&Y) scale [25] and the Movement Disorder Society (MDS)-sponsored revision of the Unified Parkinson's Disease Rating Scale (MDS-UPDRS) Part I (non-motor experiences of daily living), Part II (motor experiences of daily living, Part III (motor examination) and Part IV (motor complications) [26]. Cognitive function was assessed in all subjects using the Mini-Mental State Examination (MMSE) [20].

### MRI

Patients and controls underwent MRI of the brain with identical protocols at 3 Tesla (TimTrio, Siemens Healthcare, Erlangen, Germany) using a head coil array with 12 receiver channels. MRI gradient echo images for QSM reconstruction and calculation of R2* rate constants were acquired with the same spoiled 3D multi-echo gradient-echo sequence with six equally spaced echoes (TR/TE1/FA = 35 ms/4.92 ms/20°, inter-echo spacing = 4.92 ms, bipolar echo readout, in-plane resolution = 0.9 x 0.9 mm^2^, slice thickness = 2 mm, 64 slices, acquisition time = 4:51 minutes).

A T1-weighted 3D magnetization-prepared rapid gradient-echo (MPRAGE) sequence (TR/TE/TI/FA = 1900 ms/2.19 ms/900 ms/9°, acquisition time = 6:01 minutes) with 1 mm isotropic spatial resolution was used for segmenting deep gray matter structures and performing brain volumetry.

### Image Processing and Analysis

Physicians and physicists were both blinded to the clinical information when performing the image analyses. R2* and QSM images were reconstructed according to algorithms previously described in more detail [8,10].

In brief, images of the gradient echo sequence were linearly registered to the first echo to correct for image shifts induced by the bipolar readout gradient and R2* was then calculated pixel wise by applying a noise truncation model and mono-exponential fitting [8]. Susceptibility maps were obtained by calculating a field map from the even echoes [27] and using the homogeneity enabled incremental dipole inversion (HEIDI) method where the background phase was removed from the measured gradient echo phase data by applying the V-SHARP algorithm (maximum radius 9 mm) [10,28,29].

The T1 weighted MP-RAGE scan served to segment the bilateral caudate nucleus, globus pallidus, putamen, and thalamus in an automated manner using FreeSurfer [30]. The substantia nigra (both pars compacta and pars reticulata combined) and nucleus ruber were segmented semi-automatically. The resulting 3D models of these structures were eroded by one voxel to reduce partial volume effects and then used to mask all regions of interest on the QSM and R2* maps. Mean R2* rate constants and magnetic susceptibility (referenced to susceptibility in the cerebrospinal fluid) and the respective standard deviations were calculated for each brain structure and averaged over both hemispheres. Normalized brain volume and regional volumes of white matter, cortical and total gray matter were measured using SIENAX, as part of the FSL toolbox [31].

### Statistical Methods

All analyses were performed using the statistical software STATISTICA 10 (StatSoft Inc, Tulsa, USA). A p–value of *p* < 0.05 was considered as statistically significant. Because of the exploratory nature of our analysis with no pre-specified hypothesis, no adjustments for multiple testing were done (30).

Normal distribution of data was tested with the Kolmogorov–Smirnov test. Differences in brain volumes, R2*, and magnetic susceptibility between patients with PD and controls were tested using the paired t-test or Mann–Whitney U-test, where appropriate. According to the underlying age-dependent accumulation of iron during lifespan, correlations were corrected for effects of age [32]. Spearman correlation analyses served to investigate the relation of R2* and magnetic susceptibility with clinical and demographic data as well as brain volumes. As the differences in morphologic changes and their clinical correlations may change with disease duration, we also reassessed them after dichotomizing patients with respect to the median disease duration (3.4 years).

## Results

### Patient Characteristics and Morphology

Demographic and clinical characteristics of the study participants are listed in Table 1. Control and PD groups were comparable with respect to age and gender distribution. Morphological characteristics for patients and controls are given in Table 2. The normalized volumes of gray matter and the cortex were smaller in patients with PD compared to controls. Otherwise, patients with PD had a significantly greater ventricular volume than controls.

**Table 2:** Morphological data.

	Controls	Parkinson’s disease	Significant differences
Brain volume	1476 (73)	1463 (75)	n.s. ^a^
Gray matter volume	737 (44)	718 (40)	p<0.01 ^b^
Cortical volume	598 (37)	583 (36)	p<0.01 ^b^
White matter volume	740 (38)	745 (43)	n.s. ^a^
Ventricular volume	46 (20)	56 (23)	p<0.01 ^b^

Volumes [ml] are given as mean (standard deviation) and have been normalized to the standard brain volume;

^a^ t-test;

^b^ Mann–Whitney U-Test;

n.s. = not significant (p>0.05); Significant differences are given as group<>group (p-value).

### Group Differences as Assessed by R2* Mapping and QSM

**Fig 1:**
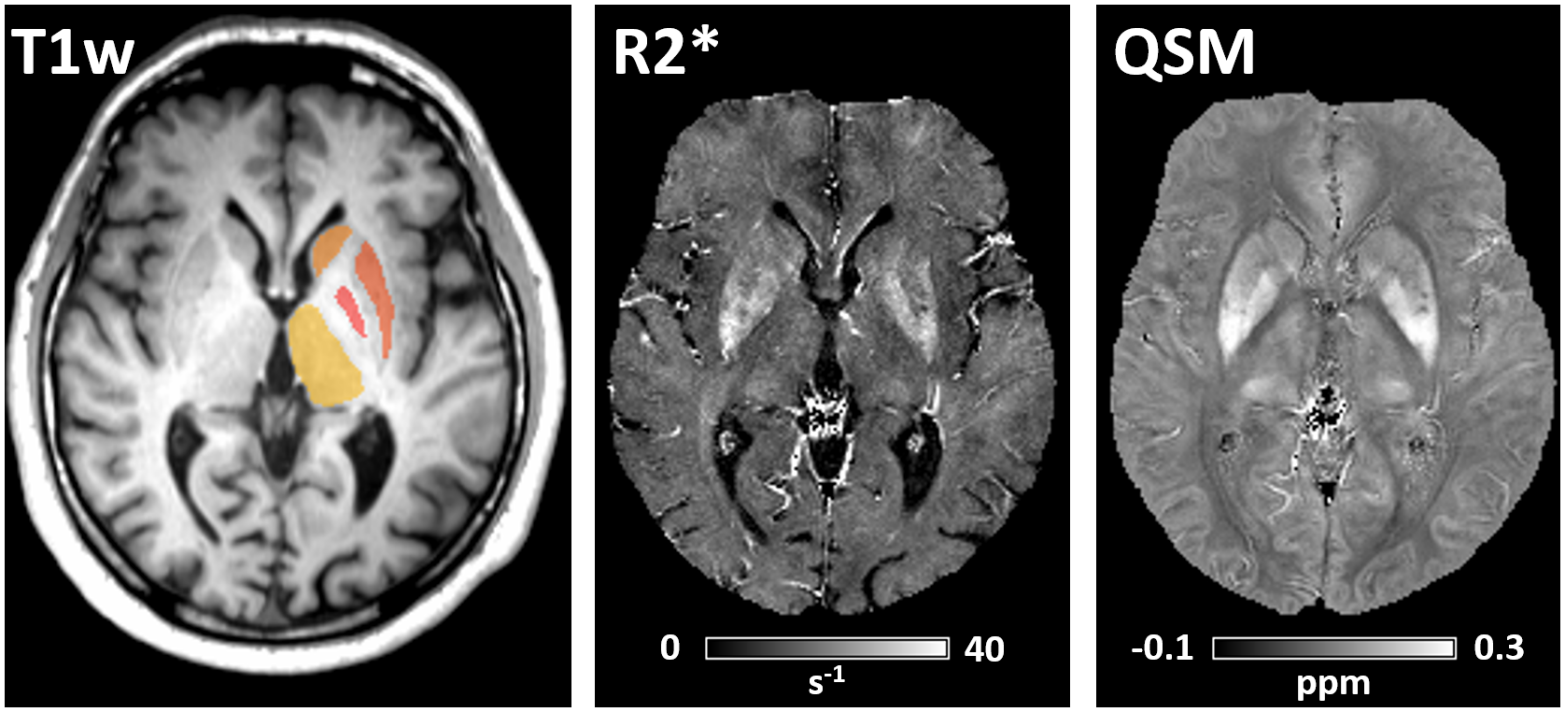
Representative T1-weighted MP-RAGE image (left) with color overlay of segmented deep gray matter structures, R2* relaxation rate constant map (center) and quantitative susceptibility map (right) of a 62-years patient with PD (MDS-UPDRS 46; Hoehn & Yahr 2). High R2* rates and paramagnetic susceptibilities are found in the basal ganglia. Note the improved gray-white matter contrast on the QSM map compared to the R2* map.

Maps of R2* and magnetic susceptibility were successfully calculated and reconstructed. Fig 1 provides representative R2* and QSM maps of a patient with PD where QSM demonstrates a higher contrast between gray and white matter than R2*, which is especially pronounced in the basal ganglia and cortical structures. Fig 1 also shows a structural T1 weighted MR image overlaid with the results of the automated segmentation of the basal ganglia and thalamus.

**Fig 2:**
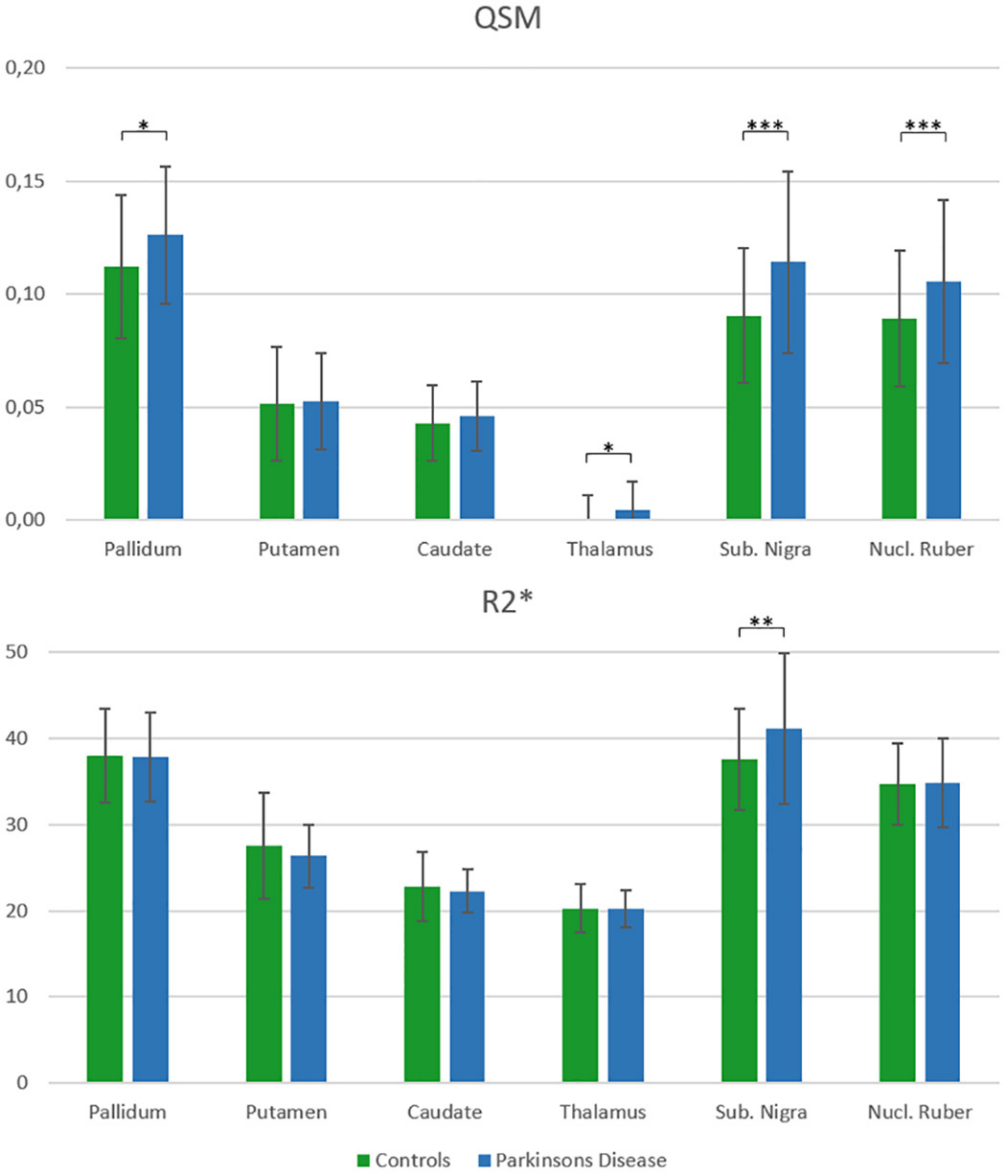
Regional mean susceptibilities and R2* (±standard deviation). Asterisks denote significant differences between controls and patients with Parkinson’s disease (*p<0.05; **p<0.01; ***p< = 0.001).

R2* rates and magnetic susceptibility values of deep gray matter structures in controls and PD patients are summarized in Table 3 and Fig 2. While both R2* relaxation rates and magnetic susceptibilities were significantly increased (i.e. more paramagnetic) in the substantia nigra of patients with PD compared to controls, a more widespread involvement of subcortical structures including the globus pallidus, thalamus and the nucleus ruber was revealed in PD by QSM.

**Table 3:** Regional magnetic susceptibility and R2* values.

R2*	Controls	Parkinson’s disease	Significant differences
Globus pallidus	38.0 (5.5)	37.8 (5.2)	n.s.^a^
Putamen	27.6 (6.2)	26.3 (3.6)	n.s.^a^
Caudate nucleus	22.8 (4.0)	22.3 (2.5)	n.s.^a^
Thalamus	20.3 (2.8)	20.2 (2.2)	n.s.^a^
Substantia nigra	37.6 (5.8)	41.1 (8.7)	**p<0.005** ^a^
Nucleus ruber	34.7 (4.8)	34.8 (5.2)	n.s.^a^
**QSM**	Controls	Parkinson’s disease	Significant differences
Globus pallidus	0.112 (0.03)	0.126 (0.03)	**p = 0.02** ^a^
Putamen	0.052 (0.03)	0.052 (0.02)	n.s.^a^
Caudate nucleus	0.043 (0.02)	0.046 (0.02)	n.s.^a^
Thalamus	0.000 (0.01)	0.005 (0.01)	**p<0.05** ^a^
Substantia nigra	0.090 (0.03)	0.114 (0.04)	**p<0.001** ^b^
Nucleus ruber	0.089 (0.03)	0.105 (0.04)	**p = 0.001** ^b^

R2* rates [s^-1^] and magnetic susceptibility [ppm] are given as mean (standard deviation) and significant differences as group<>group (p-value);

^a^ t-test;

^b^ Mann–Whitney U-Test;

n.s. = not significant (p>0.05);

When comparing right dominant to left dominant patients with PD, no regional differences of R2* and QSM were found.

### Correlations of QSM and of R2* Mapping with Clinical and Morphologic Parameters

Table 4 summarizes the results of the univariate regression analyses. Several correlations of regional R2* rate constants and magnetic susceptibilities were found for MDS-UPDRS-I and MDS-UPDRS-II. In contrast, there was no correlation of clinical and morphological parameters with MDS-UPDRS-III and MDS-UPDRS-IV. Total MDS-UPDRS was correlated with R2* and magnetic susceptibilities in the globus pallidus and the substantia nigra. The Hoehn & Yahr (H&Y) scale was correlated with nigral susceptibility and disease duration with nigral R2*. The levodopa equivalent daily dose (LEDD) was positively correlated with R2* and susceptibility in the substantia nigra. In addition and reflecting the more widespread involvement found at group level a correlation between susceptibility and LEDD was found in the globus pallidus.

**Table 4:** Univariate correlations of R2* and magnetic susceptibility with clinical parameters for patients with PD.

	H&Y	LEDD	MDS-UPDRS-I	MDS-UPDRS-II	MDS-UPDRS total	Disease duration
R2*						
Globus pallidus	-	-	0.37	0.33	0.25	-
Putamen	-	-	0.28	-	0.25	0.26
Caudate nucleus	-	-	0.25	-	-	-
Thalamus	-	-	-	-	-	-
Substantia nigra	-	0.32	0.43	0.42	0.29	0.35
Nucleus ruber	-	-	0.27	-	-	-
**QSM**						
Globus pallidus	-	0.33	0.33	0.40	0.25	-
Putamen	-	-	0.30	-	-	-
Caudate nucleus	-	-	0.31	0.29	-	-
Thalamus	-	-	-	0.27	-	-
Substantia nigra	0.29	0.40	0.31	0.37	0.27	-
Nucleus ruber	-	-	-	-	-	-

PD = Parkinson’s disease; Values denote Spearman correlation coefficient; Unified Parkinson’s Disease Rating Scale (MDS-UPDRS); H&Y = Hoehn & Yahr scale; LEDD = levodopa equivalent daily dose

Only significant results with p-values < 0.05 are shown here. No correlation with MDS-UPDRS-III and MDS-UPDRS-IV was found.

### Comparison of PD Patients with Shorter and Longer Disease Duration

Dichotomization of the PD patients with respect to disease duration (median = 3.4 years) did not reveal differences in age, MDS-UPDRS-I, and MDS-UPDRS-IV, but in LEDD (p<0.01), MDS-UPDRS-II (p<0.01), MDS-UPDRS-III (p<0.005), total MDS-UPDRS (p<0.001), and H&Y (p<0.01). We found no differences regarding regional susceptibilities, whereas R2* in the putamen (p<0.05) and in the substantia nigra (p<0.005) was increaed in patients with longer disease duration.

Clinical correlations with regional susceptibilities and R2* are shown in Table 5 for both patient subgroups. Overall, correlations were stronger in patients with longer disease duration, except for the association with LEDD. In the patient group with shorter disease duration, LEDD was correlated with magnetic susceptibilities of several gray matter regions, whereas in patients with longer disease duration a significant correlation was noted in the putamen only.

**Table 5:** Univariate correlations of R2* and magnetic susceptibility for patients with shorter and longer disease duration dichotomized by median disease duration (3.4 years).

	PD with shorter disease duration					PD with longer disease duration				
	H&Y	LEDD	MDS-UPDRS-I	MDS-UPDRS-II	MDS-UPDRS total	H&Y	LEDD	MDS-UPDRS-I	MDS-UPDRS-II	MDS-UPDRS total
R2*										
Globus pallidus	-	-	-	-	-	-	-	0.42	-	-
Putamen	-	-	-	-	-	-	-	0.38	-	-
Caudate nucleus	-	-	-	-	-	-	-	-	-	-
Thalamus	-	-	-	-	-	-	-	-	-	-
Substantia nigra	-	-	-	-	-	0.35	-	0.54	0.37	-
Nucleus ruber	-	-	-	-	-	-	-	-	-	-
**QSM**										
Globus pallidus	-	0.37	-	-	-	0.44	0.37	0.43	0.47	0.39
Putamen	-	0.39	-	-	-	-	-	0.48	-	-
Caudate nucleus	-	-	-	-	-	-	-	-	0.40	-
Thalamus	-	0.35	-	-	-	-	-	-	-	-
Substantia nigra	-	0.49	-	-	-	0.39	-	0.37	0.36	-
Nucleus ruber	-	0.42	-	-	-	-	-	-	-	-

PD = Parkinson’s disease; Values denote Spearman correlation coefficient; Unified Parkinson’s Disease Rating Scale (MDS-UPDRS); H&Y = Hoehn & Yahr scale; LEDD = levodopa equivalent daily dose

Only significant results with p values < 0.05 are shown here.

## Discussion

The present study confirms the results of recent investigations showing increased R2* and magnetic susceptibility in the substantia nigra of patients with PD compared to healthy controls [14–16,33]. Related studies have found regional susceptibility or R2* differences in single individual anatomical structures such as the putamen, caudate nucleus or the globus pallidus [17,34,35]. Extending these findings, we were able to demonstrate a more widespread involvement of the deep gray matter structures by applying QSM, including the substantia nigra, nucleus ruber, thalamus, and globus pallidus. This implies a higher sensitivity of QSM for pathological tissue changes compared to conventional relaxation rate mapping techniques with QSM being more sensitive to pathology-induced tissue changes in extra-nigral regions (Table 3). Therefore, QSM is a promising technique for the depiction of PD related tissue changes at an early stage.

This observation does not come totally unexpected, as a higher sensitivity of QSM for tissue changes was also found in other disorders, like multiple sclerosis [36,37]. Noteworthy, both R2* and QSM are derived from a conventional gradient echo sequence, which is already part of many routinely applied MR imaging protocols of the brain. Therefore, existing studies can be extended to include QSM without higher patient burden or extra measurement time.

While the higher sensitivity of QSM compared to R2* has been reiterated in this work, the underlying reasons are not entirely clear. Both measures are derived from the same gradient echo sequence and are subject to susceptibility related contributions—however, in a different manner. Ferritin-bound iron deposition increases the R2* relaxation rate and causes higher (more paramagnetic) tissue susceptibility. As shown in demyelinating disorders, pathologic changes reducing the integrity of white matter also exhibit a shift towards paramagnetic susceptibility [36]. Thus, it can be speculated that neuronal degeneration in PD would decrease the R2* rate but in parallel also lead to increased (more paramagnetic) tissue susceptibility [38]. Consequently, neurodegenerative processes underlying PD in the basal ganglia contribute additively to the effect of iron deposition, which renders QSM more sensitive to pathological tissue changes than R2* mapping. Given the current stage of understanding of this novel MR imaging contrast, this is a plausible explanation for the increased sensitivity of QSM for overall tissue pathology in PD in addition to the higher sensitivity for iron.

In this study, susceptibilities and R2* rates correlated with markers of disease severity regarding both motor (MDS-UPDRS-II, H&Y) and non-motor symptoms (MDS-UPDRS-I) of PD (total MDS-UPDRS) in several regions. This is in line with current concepts recognizing that dysfunctions within the basal ganglia induce cognitive and behavioral deficits in addition to the motor symptoms in disorders such as PD [39]. While nigral degeneration is linked to levodopa-responsive motor symptoms, such as bradykinesia and rigidity, extra-nigral pathology is thought to be involved in other motor symptoms such as tremor and postural instability, and most non-motor symptoms [40]. In our study, non-motor symptoms were only assessed by the MDS-UPDRS-I, which incorporates different aspects of non-motor symptoms. Further studies using objective tests and focused questionnaires are needed to investigate the correlation of R2*/ QSM and single non-motor symptoms such as cognition, depression, apathy, sleep problems, and bladder dysfunction. The lack of correlations between R2* / QSM and MDS-UPDRS-III may be explained by residual treatment effects at the time of motor examination after an overnight drug withdrawal, as all but eight of the treated PD patients received retarded formulations of dopamine agonists. UPDRS II, however, assesses motor performance in daily life during the past week and is thought to be a more sensitive measure for disease motor progression [33,41,42].

Both QSM and R2* mapping demonstrated certain correlations with clinical measures and in addition to R2*, QSM showed further correlations with MDS-UPDRS, LEDD and H&Y score. However, clinical correlations with R2* relaxation rates and magnetic susceptibilities were only moderate and revealed a heterogeneous pattern which might at least partially be ascribed to the inter-correlations between various symptoms.

In line with recent work, the present study found correlations between magnetic susceptibility and clinical status of the patients as assessed by the UPDRS and disease duration [17,33]. While the aforementioned studies focused on early PD, we also included patients with longer disease duration. However, the broader variance in clinical expression of the disease in the present study was not reflected in a higher correlation with QSM and R2* measures, which suggests a change in associations over the course of the disease. This is supported by our finding of quite different patterns of correlations in patients with shorter versus those with longer disease duration. While we observed significant correlations with clinical parameters in the patients with longer disease duration, magnetic susceptibilities of several deep gray matter regions correlated with the levodopa equivalent daily dose (LEDD) in the subgroup with shorter disease duration (Table 5). It may thus be speculated that QSM is able to detect tissue changes already at a level that is primarily relevant for treatment response. Thereafter and with progressing disease, the association with clinical findings intensifies. As this interpretation comes from a cross-sectional investigation, it requires longitudinal studies to confirm this hypothesis.

Our study has several limitations. The substantia nigra was not further separated in pars compacta and pars reticulata because of the rather low resolution of the clinical GRE data which prevented investigations of intra-structural spatial distributions as demonstrated in related work at 7 Tesla [43,44]. We also did not include patients with atypical parkinsonian disorders such as multiple system atrophy or progressive supranuclear palsy. However, from a clinical point of view it is of substantial interest whether QSM or R2* are capable of improving the differential diagnosis, especially at early stages, which should be subject of further studies. Although QSM images were calculated for the entire brain, we did not include white matter structures because white matter has a highly anisotropic tissue microstructure, which is consequently confounding the susceptibility values obtained [45]. Therefore, this study focused exclusively on deep gray matter. Finally, the cross-sectional design of this study prevents conclusions whether iron is directly involved in the etiology of PD or merely represents a secondary effect. Longitudinal studies are warranted to explore this further, especially in the context of currently conducted deferiprone iron-chelation-therapy studies [5,46,47].

In conclusion, we demonstrated superior sensitivity of QSM in the assessment of PD-related tissue changes in deep gray matter over R2* relaxation rate mapping with susceptibility changes also occurring in nigrostriatal dopaminergic pathways outside the substantia nigra. In addition, we were able to demonstrate correlations between QSM and motor and non-motor disease severity, levodopa-equivalent daily dose (LEDD) and H&Y score.
